# Impact of Anti-CD38 Monoclonal Antibody Therapy on CD34+ Hematopoietic Stem Cell Mobilization, Collection, and Engraftment in Multiple Myeloma Patients—A Systematic Review

**DOI:** 10.3390/ph17070944

**Published:** 2024-07-15

**Authors:** Flavia Bigi, Enrica Manzato, Simona Barbato, Marco Talarico, Michele Puppi, Simone Masci, Ilaria Sacchetti, Roberta Restuccia, Miriam Iezza, Ilaria Rizzello, Chiara Sartor, Katia Mancuso, Lucia Pantani, Paola Tacchetti, Michele Cavo, Elena Zamagni

**Affiliations:** 1 IRCCS Azienda Ospedaliero-Universitaria di Bologna, Istituto di Ematologia “Seràgnoli”, 40138 Bologna, Italy; flavia.bigi@studio.unibo.it (F.B.); enrica.manzato@studio.unibo.it (E.M.); simona.barbato3@unibo.it (S.B.); marco.talarico@studio.unibo.it (M.T.); michele.puppi@studio.unibo.it (M.P.); simone.masci2@studio.unibo.it (S.M.); ilaria.sacchetti4@studio.unibo.it (I.S.); roberta.restuccia@studio.unibo.it (R.R.); miriam.iezza@studio.unibo.it (M.I.); ilaria.rizzello@unibo.it (I.R.); chiara.sartor2@unibo.it (C.S.); katia.mancuso3@unibo.it (K.M.); lucia.pantani2@unibo.it (L.P.); paola.tacchetti2@unibo.it (P.T.); michele.cavo@unibo.it (M.C.); 2 Dipartimento di Scienze Mediche e Chirurgiche, Università di Bologna, 40138 Bologna, Italy

**Keywords:** daratumumab, isatuximab, hematopoietic stem cells, multiple myeloma, mobilization, collection, autologous stem cell transplant, plerixafor

## Abstract

This systematic review examines the available clinical data on CD34+ cell mobilization, collection, and engraftment in multiple myeloma patients treated with the anti-CD38 monoclonal antibodies daratumumab and isatuximab in clinical trials and in real life. Twenty-six clinical reports were published between 2019 and February 2024. Most studies documented lower circulating CD34+ cells after mobilization compared to controls, leading to higher plerixafor requirements. Although collection yields were significantly lower in approximately half of the studies, the collection target was achieved in similar proportions of daratumumab- and isatuximab-treated and nontreated patients, and access to autologous stem cell transplant (ASCT) was comparable. This could be explained by the retained efficacy of plerixafor in anti-CD38 monoclonal antibody-treated patients, while no chemotherapy-based or sparing mobilization protocol proved superior. Half of the studies reported slower hematopoietic reconstitution after ASCT in daratumumab- and isatuximab-treated patients, without an excess of infectious complications. While no direct effect on stem cells was observed in vitro, emerging evidence suggests possible dysregulation of CD34+ cell adhesion after daratumumab treatment. Overall, anti-CD38 monoclonal antibodies appear to interfere with CD34+ cell mobilization, without consistently leading to significant clinical consequences. Further research is needed to elucidate the underlying mechanisms and define optimal mobilization strategies in this patient population.

**Main Message**

After daratumumab and isatuximab therapy:

∘ Lower peaks of circulating CD34+ cells after mobilization;
∘ Higher use of plerixafor and longer mobilization procedures;
∘ Lower CD34+ cell collection yields;
∘ Slower hematopoietic recovery after autologous transplant.

However, clinical consequences are generally not relevant.

No correlation with number of daratumumab doses, treatment-free interval before mobilization and mobilization strategy.

Retained efficacy of plerixafor.

Possible dysregulation of CD34+ cell adhesion and homing.

## 1. Introduction

The landscape of multiple myeloma (MM) treatment has been recently reshaped by the introduction of anti-CD38 monoclonal antibodies (MoAbs) daratumumab and isatuximab. Indeed, the inclusion of these antibodies in the therapeutic backbone offers clear benefits in terms of treatment responses, minimal residual disease (MRD), and survival, as previously discussed in this issue. In particular, daratumumab has recently become the staple of first-line therapy, although concerns have emerged regarding its impact on stem cell mobilization prior to autologous stem cell transplant (ASCT).

The survival benefit of ASCT in MM has been demonstrated in randomized clinical trials after proteasome inhibitors-based triplet induction [1,2,3]. ASCT was also integral to the design of the CASSIOPEIA clinical trial, which led to the approval of the first daratumumab-based quadruplet as induction therapy in MM [4], and of the studies investigating other anti-CD38 MoAb-based therapies in the same setting [5,6,7,8,9]. For these reasons, even if its survival advantage after anti-CD38 MoAb-based induction quadruplets has yet to be definitively demonstrated and an MRD-driven approach aimed at sparing high-dose chemotherapy is currently under investigation in this setting [10], ASCT remains crucial for eligible patients who aim for long-term remission, as recommended by the most recent guidelines [11,12].

Several authors have reported reduced stem cell yields in daratumumab-treated patients, along with lower circulating CD34+ cell peaks, increased use of plerixafor (PLX), and longer duration of leukapheresis, with potential cost implications. A delayed hematopoietic recovery after ASCT has also been discussed. These observations were consistent in both clinical trials and real-world clinical practice.

The harvest of autologous CD34+ hematopoietic stem cells (HSCs) for ASCT is usually performed after induction therapy. In the past, high doses of cyclophosphamide (CY), up to 7 g/sqm, were used for both cytoreduction and mobilization. Other chemotherapeutic agents, namely, etoposide, can be added. However, with the emergence of novel anti-MM therapies, intermediate-to-low doses of CY have become the preferred approach. Indeed, these doses (1.5–4 g/sqm) retain mobilization capacity without increased toxicity [13,14]. Chemotherapy-sparing strategies, relying on granulocyte-colony stimulating factor (G-CSF), are also viable options, particularly after the approval of the anti-CXCR4 agent PLX [15]. These approaches generally allow sufficient HSC yields with reduced toxicity and possibly reduced costs [16] and have been favored in patients with cardiac and/or renal impairment, as well as in unique situations like pandemic COVID-19 [17]. The main strategies for PLX use before leukapheresis are “upfront”, i.e., planned, or “rescue”, i.e., in case of suboptimal CD34+ cell count in peripheral blood or in case of insufficient yield after the first day(s) of collection.

Protocols for mobilization vary significantly and tend to be center-specific and often tailored to individual patients. Despite these differences, however, the issue of the interference of anti-CD38 MoAbs with HSC-related outcomes has recently gained attention in an increasing number of institutions worldwide, revealing striking similarities alongside subtle distinctions. However, a review on this topic is still lacking. This review aims to provide a comprehensive overview of the existing literature, exploring the biological, clinical, and economic impact of anti-CD38 MoAb therapy on HSC mobilization, collection, and post-ASCT engraftment. The proposed associated risk factors and the efficacy of different mobilization strategies will also be discussed.

## 2. Methods

**Figure 1:**
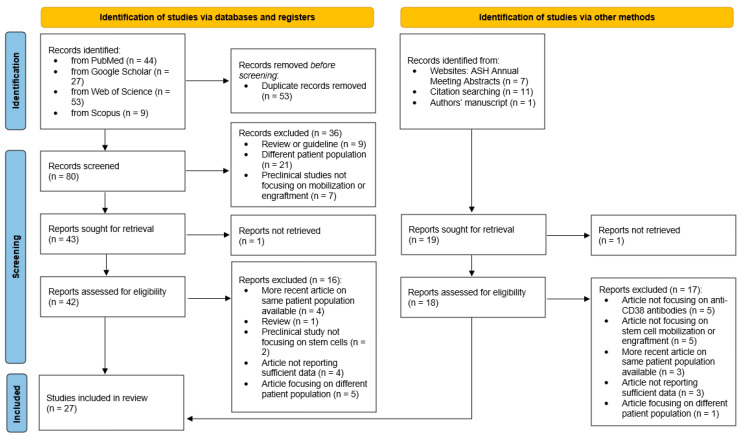
PRISMA flow diagram.

This review was carried out following the recommendations outlined in the PRISMA 2020 statement (see Figure 1 for the PRISMA flow diagram). We initially retrieved relevant publications with a PubMed and Google Scholar search on 25 February 2024. Our search string was as follows: (“daratumumab” OR “isatuximab”) AND ((“mobilization” or “mobilisation”) OR “collection” OR “apheresis” OR “engraftment” OR “CD34” OR “hematologic recovery”) AND “myeloma” and was limited to articles published in English between 2018 and February 2024. Also, Web of Science and Scopus repositories were consulted to maximize the inclusion of published data, by applying the same methods. Additionally, we reviewed the proceedings of the American Society of Hematology annual conferences from 2020 to 2023. Subsequently, we examined the references cited in the retrieved articles for further relevant information. 

After removing duplicates, two independent reviewers assessed the remaining titles for inclusion in this paper. We considered all published clinical studies that investigated outcomes related to stem cell mobilization, harvest, and ASCT involving daratumumab or isatuximab. Furthermore, we included a research article authored by our group, which was under peer review for publication at that time [18]. 

Each reviewer independently contributed to creating a dataset comparing all available data from the analyzed studies regarding induction therapies, number of planned ASCTs, collection targets, mobilization strategies, PLX use, number of circulating CD34+ cells, HSC yield, access to ASCT, hematopoietic recovery, transfusions requirements, infectious complications, and duration of hospitalization after ASCT. Additionally, we performed the Mann–Whitney U-test and Fisher’s exact test on the dataset provided by Zappaterra et al. [19], to extrapolate the same data in that study population; the results are summarized in Table S1.

The risk of bias was independently assessed by the reviewers for each study, excluding those where it was deemed to be significant. In case of disagreement, a third reviewer was involved.

## 3. Results

Our search yielded a total of 15 full-text articles and 11 conference abstracts reporting clinical data on anti-CD38 antibodies published between 2019 and February 2024 (please refer to the PRISMA flow diagram in Figure 1 for more details). Most of the articles were about daratumumab, one focused on isatuximab, and one on both MoAbs. Three articles included biological data on HSCs [19,20,21]. We also retrieved one in vitro study testing possible mechanisms of interference with stem cell mobilization and/or engraftment [22].

For the purpose of this analysis, the results from the MASTER and GRIFFIN studies, reported in the same article [23], are described as separate studies. The results of isatuximab are discussed separately.

**Figure 2:**
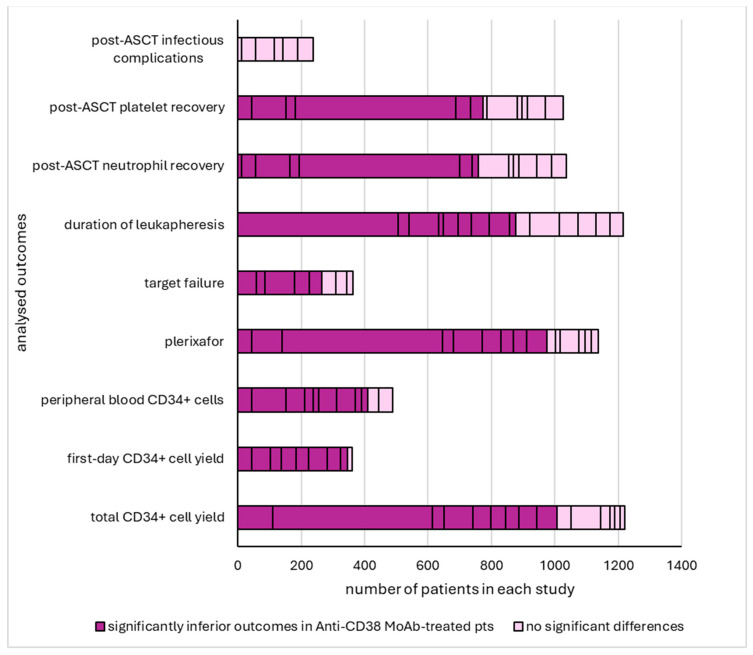
Visual summary of outcomes: Studies with and without a significant impact of daratumumab or isatuximab induction on CD34+ cell-related outcomes. Abbreviations: ASCT: autologous stem cell transplant, MoAb: monoclonal antibody, pts: patients.

Table 1 summarizes induction therapies and mobilization strategies, Table 2 mobilization and collection outcomes, and Table 3 post-ASCT hematopoietic recovery. Figure 2 displays a summary of the analyzed evidence. 

### 3.1. Characteristics of the Studies

The retrieved clinical reports include 2 case reports [24,25], a commentary on one of them [26], 3 sub-analyses of randomized clinical trials (namely, the GRIFFIN and MASTER trials [23], the CASSIOPEIA [27], and the LCI-HEM-MYE-KRdD-001 [28] trials), 16 retrospective [18,19,21,29,30,31,32,33,34,35,36,37,38,39,40,41], and 3 prospective studies [20,42,43]. Among the observational studies, 15 were monocentric [18,19,20,21,29,32,33,36,37,38,39,40,41,43,44] and 5 multicentric [30,31,34,35,42]. Nineteen studies provided a control group with patients not treated with an anti-CD38 MoAb [18,19,20,21,23,27,29,31,32,33,34,36,37,38,39,40,41,42,44].

**Table 1:** Induction therapy and mobilization protocols of the analyzed studies.

First Author, EU/SA/US [Reference]	Anti-CD38 MoAb-Treated Patients (nr)	Induction Quadruplet	Mobilization Therapy	Plerixafor Strategy	Collection Goal (CD34+ Cells × 10^6^/kg)
Studies with a non-anti-CD38 MoAb-treated control group					
Al Saleh, US [29]	12	DIRd or DVCd	G-CSF 10 μg/kg/d	ns	
Bigi, EU [18]	44	DVTd or DVCd	CY 2–3 g/sqm + G-CSF 10 μg/kg/d	rescue	3–6
Cavallaro, EU [31]	109	DVTd	CY 1–3 g/sqm + G-CSF 10 μg/kg/d	rescue	ns
Chaabra, US [23] (GRIFFIN)	95	DVRd	G-CSF	rescue or upfront	2–5
Edmisson, US [32]	58	DVRd	G-CSF	upfront	5
Fazio, EU [33]	28	DVTd	CY 2.4–3 g/sqm + G-CSF 10 μg/kg/d	rescue	ns
Hulin, EU [27] (CASSIOPEIA)	506	DVTd	CY 2–3 g/sqm + G-CSF 10 μg/kg/d	rescue	ns
Kauer, EU [40]	35	IVRd	CAD or CY 2 g/sqm + G-CSF 10 μg/kg/d	rescue	6
Lemonakis, EU [34]	92	DVTd or DVRd	CY + G-CSF	rescue	4
Luan, US [36]	16	ns	G-CSF	rescue	ns
Manjappa, US [21]	16	ns	G-CSF	ns	ns
Mina, EU [42]	57	DVTd	G-CSF 10 μg/kg/d	rescue	4
Oza, US [44]	47	ns	CdE + G-CSF or G-CSF	rescue or upfront	8–12
Papaiakovou, EU [37]	40	ns	CY 2.5 g/sqm + G-CSF 10 μg/kg/d	rescue	5
Sauer, EU [38]	58	DVTd	CAD or CY 2 g/sqm + G-CSF 10 μg/kg/d	rescue	6
Thurlapati, US [39]	43	DVRd	G-CSF 6 μg/kg/d	upfront	2.5–5
Unis, US [41]	62	ns	Ns	ns	ns
Venglar, EU [20]	20	DVCd or IKRd	CY 2.5 g/sqm + G-CSF 10 μg/kg/d	rescue	5
Zappaterra, EU [19]	20	DVTd, DVCd or DRd	CY (2–3) + G-CSF 5 μg/kg/d	rescue	6
Studies without a non-anti-CD38 MoAb-treated control group					
Bhutani, US [28] (LCI-HEM-MYE-KRdD-001)	22	DKRd	G-CSF 10 μg/kg/d	rescue	8–12
Bourlon, EU [30]	95	DVTd	CY 1.5 g/sqm + G-CSF 5 μg/kg/d	rescue	2
Chaabra, US [23] (MASTER)	116	DKRd	G-CSF 10 μg/kg/d	rescue or upfront	2–12
Crusoe, SA [43]	21	DCTd	G-CSF	rescue	2.5
Liberatore, EU [35]	47	DVTd	CY 4 g/sqm + G-CSF 5 μg/kg/d	rescue	10

Abbreviations: CAD: cyclophosphamide 2 g/smq and doxorubicin 60 mg/sqm; CdE: cyclophosphamide, etoposide and dexamethasone; CY: cyclophosphamide; DCTd: daratumumab, cyclophosphamide, thalidomide, dexamethasone; DIRd: daratumumab, ixazomib, lenalidomide, dexamethasone; DKRd: daratumumab, carfilzomib, lenalidomide, dexamethasone; DRd: daratumumab, lenalidomide, dexamethasone; DVCd: daratumumab, bortezomib, cyclophosphamide, dexamethasone; DVRd: daratumumab, bortezomib, lenalidomide, dexamethasone; DVTd: daratumumab, bortezomib, thalidomide, dexamethasone; EU: Europe; G-CSF: granulocyte-colony stimulating factor; IKRd: isatuximab–carfilzomib–lenalidomide–dexamethasone; IVRd: isatuximab, bortezomib, lenalidomide, dexamethasone; MoAb: monoclonal antibody; nr: number; ns: not specified; SA: South America; US: North America.

**Table 2:** Mobilization and collection outcomes in patients treated with daratumumab or isatuximab.

First Author [Reference]	First Day Yield (Median, CD34+ Cells × 10^6^/kg)	Total Yield (Median, CD34+ Cells × 10^6^/kg)	Circulating CD34+ Cells (Median, /µL), (b)	Plerixafor Use (%)	Target Failure (%)	Days of Apheresis (Median)
Studies with a non-anti-CD38 MoAb-treated control group						
	vs. controls	vs. controls	vs. controls	vs. controls	vs. controls	vs. controls
Al Saleh [29]	ns	ns	ns	ns	ns	ns
Bigi [18]	**3.5** vs. 5.92	6.7 vs. 8.03	**21** vs. 81	**52** vs. 20	16 vs. 16	1.9 vs. 1.7
Cavallaro [31]	ns	**5.2** vs. 8.7	26 vs. 76	50 vs. 14	ns	ns
Chaabra [23] (GRIFFIN)	ns	8.3 vs. 9.4	ns	**41** vs. 27 (d)	2 vs. 6	2 vs. 1
Edmisson [32]	**6.0** vs. 10.6	ns	57 vs. 96 (a)	ns	**14** vs. 3	1 vs. 1
Fazio [33]	ns	9 vs. 9	44 vs. 98 (b)	29 vs. 13	**32** vs. 6	ns
Hulin [27] (CASSIOPEIA)	ns	**6.7** vs. 10.0 *	ns	**22** vs. 8	ns	**1.9** vs. 1.4 *
Kauer [40]	**5.8** vs. 7.6 *	**8.8** vs. 9.7 *	80 vs. 116 *	**34** vs. 16	0 vs. 5	2 vs. 1
Lemonakis [34]	ns	**5.1** vs. 7.2 *	ns	**37** vs. 6	**24** vs. 14	**2** vs. 1 *
Luan [36]	ns	8 vs. 10	**17.2** vs. 35	94 vs. 69	ns	**2.4** vs. 1.6
Manjappa [21]	ns	7.2 vs. 8.8	ns	ns	ns	ns
Mina [42]	ns	**7.1** vs. 7.9	**19** vs. 24	**53** vs. 28	ns	2 vs. 1
Oza [44]	ns	**9.3** vs. 11.8	ns	ns	**55** vs. 27	**3** vs. 2
Papaiakovou [37]	**8** vs. 16	**10.5** vs. 16.6	ns	**42** vs. 8	**12.5** vs. 3.8	ns
Sauer [38]	**5.5** vs. 8.3	**8.4** vs. 9.6	**65** vs. 106 *	33 vs. 21	21 vs. 3	**2** vs. 1
Thurlapati [39]	4.9 vs. 6.1	6.5 vs. 6.8	43 vs. 63 (a)	95 vs. 95	ns	1 vs. 1
Unis [41]	ns	**5.3** vs. 6.7	ns	ns	ns	**1.4** vs. 1.3
Venglar [20]	ns	10.6 vs. 13.2	**63** vs. 128 (b)	28 vs. 0	39 vs. 0	ns
Zappaterra [19]	3.9 vs. 6.9	4.0 vs. 6.9	**39** vs. 64	20 vs. 5	0 vs. 0	2 vs. 1
Studies without a non-anti-CD38 MoAb-treated control group						
Bhutani [28] (LCI-HEM-MYE-KRdD-001)	ns	7.7	4.2	82	77	1
Bourlon [30]	ns	4.5	29.2	22	15	ns
Chaabra [23] (MASTER)	ns	6	ns	88	ns	2
Crusoe [43]	ns	3.9	ns	42 (c)	ns	1
Liberatore [35]	7	10.7	57	49	6	1.6
Range of median values in anti-CD38 MoAb-treated patients						
Min	3.5	3.9	17.2	20	0	1
Max	8	10.7	80 *	95	77	3

Results in bold indicate a statistically significant difference with the control group (*p* < 0.05); * mean; (a): measured after plerixafor administration; (b): peak value; (c): in the group that received 16 daratumumab doses; (d): in the subgroup with a rescue plerixafor strategy. Abbreviations: MoAbs: monoclonal antibodies; ns: not specified.

**Table 3:** Hematopoietic recovery after autologous stem cell transplant in anti-CD38 MoAb-treated vs. nontreated patients.

First Author [Reference]	Time to Neutrophil Recovery in Anti-CD38 MoAb-Treated vs. Control Patients, Median (Days)	Time to Platelet Recovery in Anti-CD38 MoAb-Treated vs. Control Patients, Median (Days)
Al Saleh [29]	**19** vs. 16	18 vs. 17
Bigi [18]	**12** vs. 11	**14** vs. 12
Cavallaro [31]	**13** vs. 11	**13** vs. 11
Chaabra [23] (GRIFFIN)	12 vs. ns	13 vs. ns
Crusoe [43]	11 vs. 11	12 vs. 11
Fazio [33]	**14** vs. 11	**15** vs. 14
Hulin [27] (CASSIOPEIA)	**14.4** vs. 13.7	**14.9** vs. 13.6
Luan [36]	12.1 vs. 12.3	14.6 vs. 13.7
Manjappa [21]	12 vs. 12	13 vs. 12
Mina [42]	**13** vs. 15	**14** vs. 16
Oza [44]	11 vs. 11	14 vs. 13
Papaiakovou [37]	**11** vs. 10	**12** vs. 10
Venglar [20]	**12** vs. 11	**13** vs. 12
Zappaterra [19]	9.5 vs. 10	10.5 vs. 11

Results in bold indicate a statistically significant difference with the control group (*p* < 0.05). Abbreviations: MoAb: monoclonal antibody.

### 3.2. Study Populations

In total, 1613 patients treated with daratumumab were analyzed, with a median of 47 patients in each study (range 9–506) [9,27]. These were mostly patients with newly diagnosed MM, although in the study by Luan et al., 62.5% of patients were undergoing mobilization for salvage transplant, with a mean of 1.9 prior lines of therapy [36]. When specified, induction therapy most frequently consisted of the combination of daratumumab, bortezomib, thalidomide, and dexamethasone (D-VTd); seven studies included lenalidomide [23,28,29,32,34] and four cyclophosphamide [18,19,20,43]. In most studies, patients received the canonical four to six induction cycles before mobilization, while one described a longer induction with eight cycles of daratumumab, carfilzomib, lenalidomide, and dexamethasone [28].

### 3.3. Mobilization Regimens and Apheresis Targets

A total of 10 studies employed a chemotherapy-free approach using G-CSF alone in the majority of patients [21,23,28,29,32,36,39,42,43], while 13 incorporated CY, at least in younger and fit patients [18,19,20,27,30,31,33,34,35,37,38,40,44], typically administered at a dose ranging between 1 and 3 g/sqm. Notably, one study used a higher dosage of 4 g/sqm [35] and others combined CY with etoposide [44] or doxorubicin [38,40]. As for G-CSF, it was commonly administered at either 5 or 10 mcg/kg, while one report utilized a dosage of 6 mcg/kg [39].

PLX was most often used as a rescue strategy in case of low levels of circulating CD34+ cells or poor initial collection. In three studies, PLX was employed upfront after mobilization with G-CSF only [32,39,44], while in the MASTER and GRIFFIN studies, both strategies were used [23].

The CD34+ target ranged from 2 to 12 × 10^6^ CD34+ cells/kg. This variability was influenced by the collection goal for each ASCT, which was usually 2–3 × 10^6^ CD34+ cells/kg, but sometimes higher, and by the number of planned ASCTs. While most studies considered one or two ASCTs, in three of them the collection target was for three transplants [28,38,40]. 

### 3.4. CD34+ Cell Yield

In daratumumab-treated patients, the total CD34+ cell yield ranged from 3.98 × 10^6^ CD34+ cells/kg [19] to 10.68 × 10^6^ CD34+ cells/kg [35], likely influenced by the different collection targets. The yield on the first day of leukapheresis was not influenced by the collection goal, but could be affected by the timing of PLX, and ranged from 3.48 × 10^6^ CD34+ cells/kg [18] to 7.99 × 10^6^ CD34+ cells/kg [37].

Across 14 studies, daratumumab-treated patients exhibited a numerically lower total HSC yield compared to controls [18,19,20,21,23,27,31,34,36,37,38,41,42,44]. However, statistical significance was demonstrated in only eight of these studies [27,31,34,37,38,41,42,44]. Accordingly, daratumumab correlated with inferior stem cell yields in multivariate [34,38] or univariate [32] analysis in three studies. On the other hand, two studies found no difference between daratumumab and non-daratumumab groups of patients [20,33].

The number of CD34+ cells collected on the first day of leukapheresis was significantly inferior in seven studies in daratumumab patients compared to controls [18,19,32,37,38,39,44]. Daratumumab had no impact on first-day harvest in univariate [39] and multivariate [21] analyses in two studies.

### 3.5. Circulating CD34+ Cells

Across all ten studies comparing circulating CD34+ cells in peripheral blood between daratumumab-treated patients and controls, the former consistently exhibited lower levels [18,19,31,32,33,36,38,39,42]. Statistically significant differences were observed in all but one [39] of these studies.

Peripheral blood CD34+ cell levels were typically assessed on the planned first day of leukapheresis before PLX (specifically, on the 4th day of G-CSF and on day +11 after CY) and ranged between 17/mcL [36] and 65/mcL [38] (mean). Circulating HSC levels were lower in daratumumab patients also in the studies reported by Venglar at al. [20] and Fazio et al. [33] that considered azimuth values.

Poor mobilizers were generally defined as patients with less than 20 CD34+ cells/mcL in the peripheral blood on the first planned day of apheresis, and their frequency ranged from 25% [19] to 54% [42] in daratumumab-treated patients. Their incidence in control patients (6–23%) ([31,42], respectively) aligned with the existing literature [45].

Even though the mean HSC concentration was lower in daratumumab patients and the PLX requirement higher, due to <20/mcL levels of circulating CD34+ cells , Sauer et al. found a higher proportion of patients not reaching the 10/mcL threshold in the control group [38].

Zappaterra et al. found that a CD34+ cell count of 54.49/mcL was associated with a higher probability of achieving the collection goal of 6 × 10^6^ CD34+ cells/kg, with a sensitivity of 82% and specificity of 76% [19].

Three articles described a significant delay in the beginning of HSC collection in patients treated with daratumumab because of the low peripheral blood CD34+ levels on the first day of planned leukapheresis [20,33,38].

### 3.6. Plerixafor

Most studies employed a “rescue” PLX strategy, and PLX was used in cases of low circulating CD34+ counts or insufficient yields after the initial apheresis procedure(s). However, Lemonakis at al. [34] and Luan at al. [36] restricted its use to cases of low CD34+ cell concentration, while Zappaterra et al. [19] only allowed it in case of target failure. In contrast, Liberatore et al. adopted a risk-adapted approach, administering PLX also in patients with a body weight/circulating HSC ratio > 2 kg/mcL [35].

The rates of rescue PLX use in daratumumab-treated patients varied from 20% [19] to 94% [36], consistently exceeding the rates in control groups across studies [18,19,20,23,27,31,33,34,36,37,38,41,42], often with statistical significance [18,23,27,34,37,41,42].

Thurlapati et al. employed an “upfront” PLX strategy but permitted additional doses for patients with low first-day collections. The daratumumab and control groups showed no statistically significant difference in PLX requirements, despite a higher median number of PLX doses and a greater proportion of patients requiring multiple doses in the daratumumab group [39].

Three studies compared the efficacy of PLX between daratumumab-treated patients and controls [18,38,42]. They found that PLX remained effective in increasing circulating CD34+ cells [18,38,42] and enhancing the collection yield [18,38].

### 3.7. Duration of Leukapheresis

The duration of leukapheresis procedures in patients treated with daratumumab varied across studies. The median number of daily leukaphereses in daratumumab-treated patients generally ranged from one to two. In one study by Oza et al., the median number was four, but this was likely influenced by the high collection goal of 8–12 × 10^6^ CD34+ cells/kg [44]. 

In most studies, the duration of leukapheresis was significantly higher in daratumumab-treated patients compared to controls [19,27,34,36,37,38,41,44]. In a few other studies, the difference was only numerically higher [18,20,23,32,42], while Cavallaro et al. found no difference between the two groups [31]. In the study by Zappaterra et al., daratumumab correlated with the probability of undergoing multiple aphereses in multivariate analysis [19].

In the study by Papaiakovou et al., the median total duration of leukapheresis was 515 min in the daratumumab group and 426 min in the control group (*p* < 0.001) [37]. Additionally, collection volumes were higher in daratumumab patients in two studies [33,37].

### 3.8. Target Failure

The target failure rate varied significantly and was influenced by the collection goal. In daratumumab-treated patients, it ranged from 2% [23] to 77% [28]. Five studies reported a significantly higher target failure rate in daratumumab-treated patients [32,33,34,37,44]. Two studies found no significant difference with the control group [18,20]. 

Overall, the proportion of patients failing to collect the minimum institutional threshold for a single ASCT ranged from 3% [32] to 15% [30]. Significantly higher failure rates were observed only in the study by Papaiakovou et al., where the threshold was set at 5 × 10^6^ CD34+ cells/kg [37]. Interestingly, in Mina et al., the proportion of patients achieving an optimal (>4 × 10^6^ CD34+ cells/kg) and a suboptimal harvest (2–4 × 10^6^ CD34+ cells/kg) was similar between the daratumumab and control group [42].

The failure rate for two ASCTs ranged from 14% [39] to 90% [28] and was significantly higher in daratumumab patients in three studies [34,37,39], while only numerically higher in two others [18,20].

The proportion of daratumumab patients undergoing a second mobilization attempt varied widely from 0.4% [27] to 18% [33], with fluctuating rates of success. Most studies found no significant difference in rates of second mobilizations between daratumumab and control patients [18,23,27,32,44].

### 3.9. Hematopoietic Reconstitution after ASCT

The definitions of neutrophil and platelet recovery varied across studies. Platelet engraftment was significantly slower in daratumumab-treated patients in seven studies [18,20,27,31,33,37,44], while no significant differences were reported in four other studies [19,21,23,29,36]. Similarly, neutrophil recovery was significantly slower in daratumumab patients in seven studies [18,20,27,29,31,33,37], but not significantly different in five [19,20,21,23,36,44]. One study reported faster platelet and neutrophil recovery in the daratumumab group [42]. Overall, the delay in hematopoietic engraftment was typically 1 or 2 days, but all patients achieved hematopoietic recovery.

In terms of transfusion requirements, one study found that daratumumab patients had a higher need for platelet transfusions (median of 4 units vs. 2 units, *p* < 0.001) [37]. Additionally, in two studies, daratumumab-treated patients received more red blood cell transfusions [18,37], while in two other studies, transfusion rates were similar between the daratumumab and control groups [20,33].

The rates of neutropenic fever were comparable between daratumumab and control patients across all studies [18,29,31,33,37,44]. However, Papaiakovou et al. reported longer durations and need for more lines of antibiotic therapy, higher incidence of septic shock, and prolonged hospitalization in the daratumumab group. This did not translate into higher day +100 mortality rates. The authors suggested that this excess risk was unlikely to be solely explained by the slight delay in neutrophil recovery. Instead, they hypothesized that daratumumab might worsen immunosuppression through hypogammaglobulinemia and lymphodepletion [37]. Another study found no significant differences in severe infections, duration of antibiotic therapy, or length of hospitalization between daratumumab-treated patients and control patients [18]. 

### 3.10. Reports on Isatuximab

Only two clinical reports on isatuximab were retrieved. In a single-center retrospective report, Kauer et al. compared 79 patients treated with isatuximab, bortezomib, lenalidomide, and dexamethasone to patients treated with the same regimen without isatuximab or with elotuzumab. Following CY and doxorubicin mobilization, despite comparable levels of pre-apheresis circulating CD34+ cells across the three groups, a significantly higher PLX requirement was observed in isatuximab patients. First-day and total HSC yields were lower and the median number of leukapheresis sessions were higher compared to non-isatuximab-treated patients. However, the proportion of patients meeting the collection goal for three ASCTs was similar in the three groups [40].

In another single-center prospective study, Venglar et al. analyzed the outcomes from 11 patients treated with isatuximab, carfilzomib, lenalidomide, and dexamethasone; 9 patients treated with daratumumab, bortezomib, CY, and dexamethasone; and 14 patients treated with VTd [20]. Outcomes were similar between isatuximab- and daratumumab-treated patients (and inferior to VTd patients) in terms of circulating CD34+ cells, collection yields, number of days of leukapheresis, and post-ASCT hematopoietic reconstitution. The total HSC yield was inferior after isatuximab than after daratumumab (4.88 vs. 10.56 × 10^6^ CD34+ cells/kg, *p* = 0.025). However, the association with lenalidomide during induction and the small sample size could have influenced this result [20]. 

### 3.11. Related Factors: Mobilization Strategies

Studies employing chemo-mobilization and those using G-CSF alone seemed to demonstrate similar differences between daratumumab-treated patients and controls, in terms of collection yields, target failure, or use of PLX. Notably, in daratumumab-treated patients, no difference was found upon 2 g/sqm vs. 3 g/sqm of CY [18]. However, Liberatore et al. reported impressive collection yields in daratumumab patients with 4 g/sqm of CY, with a median harvest of 10.68 × 10^6^ CD34+ cells/kg, though the use of PLX remained comparable to other studies with lower CY doses or without chemotherapy [35].

When examining circulating CD34+ cell levels, studies employing chemo-mobilization or upfront PLX following G-CSF mobilization consistently showed higher levels compared to G-CSF alone. After G-CSF-only mobilization and before PLX, median levels were 17.2/µL [36], 19/mcL [42], and 4.2/mcL [28], while after upfront PLX, they increased to 43/mcL and 57/mcL [32,39]. Additionally, in the study reported by Cavallaro et al., all (12%) patients receiving G-CSF-only mobilization, due to older age or renal impairment, turned out to be poor mobilizers [31]. Following chemo-mobilization, HSC levels ranged from 21/mcL (median) [18] to 65/mcL (mean) [38]. Notably, these values were particularly high in the two studies that employed high doses of chemotherapy, reaching a median of 57/mcL CD34+ cells on day +11 after CY 4 g/sqm [35], and a mean of 65/mcL on day +13 after CY-adriamycin-dexamethasone [38]. Despite these promising levels, the rates of poor mobilizers remained elevated, being 32% and 33%, respectively [35,38]. Also, patients receiving intermediate-dose CY only because of cardiovascular comorbidities (13%) had a significantly higher need for PLX compared to those who received full chemotherapy [38].

G-CSF is approved for HSC mobilization at varying doses by regulatory agencies (e.g., 5 mcg/kg/day after chemotherapy and 10 mcg/kg/day when used alone in Europe, against 10 mcg/kg/day in either situation in the United States). Notably, two reports from the same German institution employed chemo-mobilization and G-CSF at 5 mcg/kg/day in triplet-treated patients (following per approved dose) and at 10 mcg/kg/day in anti-CD38 MoAb-treated-patients [38,40]. However, an Italian study found that prolonging G-CSF administration over more days, thereby increasing the cumulative dose, did not increase circulating CD34+ cells [33].

### 3.12. Related Factors: Plerixafor Strategy

In the paper by Chhabra et al., a comparison was made between the upfront and rescue strategy in the MASTER and GRIFFIN trials after G-CSF mobilization. The median total HSC yield was numerically higher in cases of upfront PLX both in daratumumab- and bortezomib–lenalidomide–dexamethasone-treated patients, although the difference was not significant [23].

### 3.13. Related Factors: Daratumumab Cumulative Dose and Timing

A case report published by Seth et al. (Mayo Clinic) described a 73-year-old patient who experienced two failed mobilization attempts after receiving 20 weekly daratumumab doses during five daratumumab-bortezomib–lenalidomide–dexamethasone cycles. Remarkably, the concentration of daratumumab in the patient’s peripheral blood reached as high as 1250 mcg/mL. However, after daratumumab clearance from the peripheral blood, 8 months later, the patients successfully underwent a third mobilization attempt. Despite initially low levels of circulating CD34+ cells, after G-CSF mobilization and PLX rescue, the patient harvested 3.18 × 10^6^ CD34+ cells/kg in four apheresis procedures and proceeded to ASCT [25]. Accordingly, the LCI-HEM-MYE-KRdD-001 study reported a very high failure rate when patients underwent mobilization after seven or eight cycles of daratumumab, carfilzomib, lenalidomide, and dexamethasone (equivalent to 18–20 daratumumab doses). The authors recommended collecting HSCs after no more than 3–4 cycles of this regimen [28]. 

Two studies compared collection outcomes between patients receiving 12 and 16 daratumumab doses (equivalent to the canonical four or six induction cycles). No statistically significant differences were found in terms of total collection yield, PLX use, and engraftment. Additionally, longer daratumumab-free intervals before mobilization did not correlate with better collection outcomes [18,43]. Furthermore, receiving more than four daratumumab, bortezomib, lenalidomide and dexamethasone cycles did not impact the first-day harvest in another study [39].

### 3.14. Other Related Factors

In addition to daratumumab, older age [19,34,38], previous radiotherapy [19,34], and the use of alkylators [32,34] or lenalidomide [32] as part of induction were related to inferior HSC yields in some studies. However, conflicting results about the same variables were observed across other studies [18,19,34,39].

In a univariate analysis specifically conducted on daratumumab-treated patients, no significant impact of patient- or disease-related factors on collection outcomes or PLX use was found [18]. Additionally, response to therapy did not correlate with HSC yields in three separate studies [18,19,39].

Cavallaro’s findings indicate that patients who experienced hematologic toxicity during induction had a higher likelihood of being poor mobilizers [31]. In Fazio et al., daratumumab-treated patients exhibited lower platelet and neutrophil counts before mobilization compared to controls, suggesting a prolonged hematologic toxicity [33]. Interestingly, premobilization platelet count did not impact target failure in the study by Zappaterra et al. [19].

### 3.15. Molecular Mechanisms

As CD38 is commonly expressed on many subsets of CD34+ cells, the first proposed mechanism of anti-CD38 MoAb-mediated impairment on mobilization and collection is direct toxicity on HSC.

Ma et al. investigated daratumumab’s effect on CD34+ cells in vitro and demonstrated a low complement-mediated cytotoxicity. The authors linked this finding to the low density of CD38 surface expression on CD34+ cells compared to MM cells. Additionally, they observed that daratumumab did not impair HSCs’ colony-formation capacity [22]. 

Similarly, Manjappa et al. found no significant difference in the levels of BFU-E (burst forming units-erythroid) and CFU-GM (colony forming units-granulocyte macrophage) in the apheresis products of daratumumab- and non-daratumumab-treated patients [21]. In the study by Zappaterra et al., BFU-E were reduced after daratumumab induction, while CFU-GM were not significantly decreased [19].

Venglar et al. assessed the concentration of different progenitor subsets in the apheresis products after daratumumab-based, isatuximab-based, and VTd induction. CD38-low multipotent progenitors were relatively increased after daratumumab and isatuximab compared with VTd, while CD38+ erythro-myeloid progenitors, lympho-myeloid primed progenitors, and granulocyte-monocyte progenitors were decreased. Interestingly, the fraction of CD38-high B-lymphocyte progenitors was similar in the three groups, both in the apheresis products and in the bone marrow aspirates on the day before ASCT. In the same aspirates, the bone marrow fraction of CD34+ cells was globally decreased in the isatuximab and daratumumab group. The authors concluded that a direct toxicity on CD38+ progenitor is likely not the main mechanism of mobilization impairment after anti-CD38 MoAb-based induction. Instead, this phenomenon could be explained by a dysregulation of adhesion and homing capacity of HSCs. In their study, adhesion molecules were significantly upregulated by RNA-sequencing in CD34+ cells harvested from daratumumab and isatuximab patients, namely, JCAD, NRP2, MDK, ITGA3, and CLEC3B (Junctional Cadherin 5-Associated Protein, Neuropilin 2, Midkine, Integrin subunit Alpha 3, and C-Type Lectin Domain Family 3 Member B). JCAD and CLEC3B were also significantly upregulated after incubation of CD34+ cells from VTd-treated patients with isatuximab [20].

## 4. Discussion

The integration of anti-CD38 MoAbs into the treatment landscape for MM has raised concerns about their potential impact on HSCs. Since 2019, numerous real-life retrospective clinical data have been published on this matter, complemented by few preclinical experiences. A critical review of the literature underscores their interference on stem cell harvest, although the limited evidence available, mostly coming from smaller single-center studies, makes only a narrative, qualitative approach possible.

The vast majority of studies focused on daratumumab; despite fewer studies, isatuximab appeared to exhibit similar trends in terms of HSC mobilization. Given the limited available data, it is prudent to consider patients treated with either drug to be equally at risk of mobilization failure, unless further evidence suggests otherwise. Further studies may shed light on any nuanced differences between isatuximab and daratumumab in this context.

Most studies reported lower levels of circulating CD34+ cells in the peripheral blood after mobilization compared to controls, resulting in higher use of PLX. Collection yields of CD34+ cells were significantly inferior to the control groups in approximately half of the controlled studies. However, the collection target was reached in a similar proportion of patients compared to the control groups and patients treated with anti-CD38 MoAbs had comparable access to ASCT. Approximately half of the analyzed studies reported slower neutrophil and platelet recovery in anti-CD38 MoAb-treated patients, while the other half did not observe significant delays. In most studies, the delayed neutrophil engraftment did not lead to an excess of infectious complications.

In general, the available data suggest that anti-CD38 MoAbs impact HSC mobilization from the bone marrow. However, this interference does not consistently have clinically significant implications. On the other hand, although no formal cost analysis has been conducted, it is reasonable to infer that the higher PLX requirements and the often prolonged leukapheresis procedures may incur higher expenses.

Given the continued need for ASCT and the challenges posed by anti-CD38 MoAbs, optimizing HSC mobilization strategies has become increasingly urgent. In the following paragraphs, we will summarize the analyzed results in terms of timing, mobilizing agents, and collection targets.

Extensive daratumumab (and lenalidomide) exposure was associated with reduced collection efficiency in two papers, suggesting that mobilization should be performed no later than the standard four or six recommended induction cycles [25,28]. Receiving six induction cycles vs. four did not significantly affect HSC collection in two separate studies [18,43]. 

Similarly, the therapy-free interval was relevant in the case report by Seth et al., where daratumumab clearance from the peripheral blood required 8 months after heavy exposure [25]. Conversely, the daratumumab-free interval did not impact mobilization and collection after four or six induction cycles [18,43]. Anyway, considering daratumumab’s half-life of 23 days [46], a 3-week washout interval can be suggested [23,26]. 

Regarding the optimal mobilization strategy, the matter is more challenging. International recommendations on mobilization therapy date back to 2009 for the International Myeloma Working Group (IMWG) [47,48] and to 2014 for the European Group for Blood and Marrow Transplantation (EBMT) [49]. The IMWG recommended adding CY or PLX only in patients older than 65 years or who have received more than four cycles of lenalidomide. In contrast, the EBMT emphasized reliance on local guidelines to choose between G-CSF-only or chemotherapy-based mobilization strategies. In general, since different approaches allowed for sufficient HSCs yields in the pre-daratumumab era, there was no push for standardization, and institutions preferred different strategies based on long-established clinical practice and PLX availability.

Chemotherapy-based protocols generally produce higher levels of circulating CD34+ cells in the peripheral blood. A multicentric study by Zannetti et al. involving 422 non-daratumumab patients revealed significantly elevated CD34+ cell counts, lower use of plerixafor, and higher HSC cell yields following mobilization with CY at a dose of ≥3 g/sqm compared with CY at 2 g/sqm or no chemotherapy [50]. While high-dose chemotherapy carries inherent risks of hematologic toxicity, neutropenic fever, transfusion requirements, nausea, and hair loss, intermediate to low doses of CY are generally considered safe. The study by Zannetti et al. reported very low toxicity rates across all groups [50], while a slight excess of neutropenic complications was noted in a study employing higher doses of CY in daratumumab-treated patients [35].

Regarding costs, some American and European studies have shown higher costs for mobilization with G-CSF and PLX compared with chemotherapy and G-CSF (without PLX) in non-daratumumab-treated patients, associated with higher or comparable efficacy [51,52,53]. Interestingly, an Italian cost-effectiveness analysis favored a G-CSF-only approach compared with chemotherapy plus G-CSF when followed by on-demand PLX [16].

As data on mobilization strategies are not definitive in the non-daratumumab setting, we could expect the same after anti-CD38 MoAbs-based inductions. Analyzed studies employing chemo-mobilization did not demonstrate strikingly superior outcomes among daratumumab-treated patients compared to chemotherapy-free approaches, suggesting that chemotherapy alone may not overcome daratumumab’s interference with HSC mobilization.

An Italian real-life multicentric study is currently evaluating HSC-related outcomes in daratumumab-treated patients. The preliminary findings on 105 patients compared to 43 historical controls were presented at the latest EBMT meeting. In line with the published evidence, daratumumab-treated patients harbored more challenging mobilization procedures with inferior HSC yields; however, most patients still had access to ASCT. Interestingly, the total CD34+ cell yield was not influenced by the dose of mobilizing CY and G-CSF [54].

Further investigations are warranted to identify the best mobilization strategy for daratumumab-treated patients. The randomization between different mobilization therapies in the setting of clinical trials investigating anti-CD38 MoAb-based induction quadruplets setting could provide valuable insights.

The optimal approach to the use of PLX also remains a topic of interest. Chhabra et al. favored an upfront strategy following G-CSF-only mobilization. This approach ensures reliability without strict monitoring of circulating CD34+ cells, although the authors stated that collection outcomes were comparable with either strategy [23]. Algorithms for the use of PLX have been proposed in the pre-daratumumab era, essentially based on peripheral blood CD34+ cell counts, and aim to reduce mobilization costs. Costa et al. suggested a CD34+ cell threshold of 14/µL if the collection goal is 3 × 10^6^ CD34+ cells/kg and 25/µL if the goal is 6 × 10^6^ CD34+ cells/kg [55]. Shah et al. demonstrated that raising the threshold to 40 CD34+ cells/µL could save 26 apheresis days per 100 patients, with similar overall costs [56]. A more extensive incorporation of PLX into mobilization protocols could hold the key to achieving satisfactory HSC yields in this patient population. However, the high cost of the drug necessitates a detailed cost-effectiveness analysis to help define the optimal algorithm for PLX use in this context. Certainly, a cost analysis should take into consideration the organization of healthcare and the social context and could lead to different outcomes in different countries.

A better understanding of the underlying mechanisms of anti-CD38 MoAbs toxicity on HSC may help choosing the optimal approach. Some authors hypothesized a prolonged hematopoietic toxicity by daratumumab, which could translate into poor mobilization and slower engraftment. In the study by Cavallaro et al., patients who experienced hematologic toxicity during induction had inferior CD34+ cell yields [31]. Venglar et al. reported lower rates of CD34+ cells in the bone marrow of anti-CD38 MoAb-treated patients on the day before ASCT [20]. In addition, in vitro experiments and observational studies failed to demonstrate a significant toxicity of daratumumab on CD34+ cells [19,21,22]. Similar results were observed with isatuximab in vitro [57], suggesting that direct cytotoxicity is not the main mechanism of impairment of HSC-related outcomes. Another intriguing mechanism proposed is the dysregulation of HSC adhesion and homing capacity, as illustrated by Venglar et al. [20]. This aligns with findings in chronic lymphocytic leukemia, where CD38 expression enhances adhesion and migration in malignant cells, while CD38 blockade inhibits these properties [58,59]. The retained efficacy of PLX in daratumumab-treated patients, as demonstrated in the analyzed clinical reports [18,38,42], supports the hypothesis that anti-CD38 MoAb toxicity primarily affects cell adhesion. Furthermore, reduced chemotactic properties could potentially explain the observed delay in hematopoietic reconstitution after ASCT. Further studies are needed to further elucidate the exact mechanisms and their precise clinical impact. This could help identify the best approach to overcome this peculiar toxicity on HSCs.

Beyond peripheral blood CD34+ cell count, other traditional risk factors associated with poor mobilization should be considered. These include previous exposure to radiotherapy, alkylating chemotherapy or lenalidomide, older age, and high bone marrow infiltration. In addition, when anti-CD38 MoAbs are introduced into the equation, the results from analyzed studies diverge concerning these risk factors and further data are needed to fully understand their role. While patient age, organ function, and prior cytotoxic therapy exposure should inform the choice of using chemotherapy for HSC mobilization, additional data are essential to precisely assess the impact of these risk factors on stem cell collection.

Lenalidomide likely represents the second most important factor at play. In one of the analyzed reports, it was associated with a greater reduction in circulating and harvested HSCs compared to daratumumab [32]. The effect of lenalidomide on stem cell mobilization is thought to be dose-dependent [60], although this remains a matter of debate [61,62], and to be related to myelotoxicity [60], albeit the mechanism underlying myelotoxicity remains unclear. Lenalidomide enhances natural killer T-cell activity in vitro and in vivo in MM patients and induces the secretion of interferon gamma, which inhibits hematopoiesis [63]. Additionally, there are likely other immunomodulatory anti-angiogenic effects at play.

The comparability of published studies on mobilization in daratumumab-treated patients has been hindered by the variability in collection targets, encompassing the number of planned ASCTs and the specific collection goal for each ASCT.

The development of novel anti-MM agents has restricted the indications for multiple ASCTs as first-line consolidation as well as at relapse. As a consequence, only a minority of patients has a collection target for two or three transplants nowadays.

Traditionally, the minimal HSC dose required for safe hematopoietic recovery after each ASCT has been set to 2 × 10^6^ CD34+ cells/kg, although higher HSC doses correlate with faster engraftment [64]. The possible impact of daratumumab on post-transplant hematopoietic recovery raises questions about whether higher HSC doses are necessary in this patient population. 

Studies with higher CD34+ cell targets demonstrated similar delays in hematopoietic reconstitution compared to those with lower thresholds. Typically, these delays amounted to one or two days and occasionally translated into higher transfusion requirements. An increase in infectious complications was not noted in the retrieved studies, but the clinical impact of slower neutrophil recovery needs to be investigated further.

Notably, a single study described higher-grade infectious complication in the daratumumab group, that the authors correlated to B-cell toxicity induced by daratumumab [37]. None of the retrieved studies analyzed lymphocyte recovery after ASCT, and in the study by Venglar et al., B-lymphocyte precursors were present in similar fractions in the bone marrow of patients treated with daratumumab, isatuximab, or VTd before autologous HSC reinfusion [20]. However, if further substantiated, this finding could favor CY-sparing mobilization approaches, which could ensure higher lymphocyte counts in the apheresis products, facilitating faster lymphocyte recovery [65,66].

This analysis has some limitations. First, most of the data came from small, single-center studies. In addition, the variability of induction therapies, mobilization strategies, and collection targets hindered the comparability of published studies, as did the different ways in which data were reported. As a result, our approach had to be predominantly qualitative. 

Collectively, the available data clearly indicate that mobilization is problematic in patients treated with daratumumab, and the same will likely occur with isatuximab once the drug is approved for frontline use. In this view, patients should be informed about the high probability of the use of PLX and the need for multiple days of leukapheresis, or second mobilization, when necessary. Also, patients should be aware of the slightly delayed hematopoietic recovery after ASCT, which may result in higher than usual transfusion requirements and, possibly, longer hospitalization. On the other hand, these issues should also be discussed with the transfusion centers and the hospital pharmacies to ensure an optimal management of resources. Nevertheless, despite the impact on HSC-related outcomes, the outstanding benefits of anti-CD38 MoAbs in terms of response and survival should not discourage their use in clinical practice. However, the identification of optimal mobilization strategies remains crucial. In this sense, the currently available literature does not favor one approach over another, as many studies report sufficient collection yields using diverse mobilization strategies. Importantly, the choice should take into consideration the established practices, expertise, reimbursement policies, and available resources in different settings and countries. Additional real-world data and, ideally, randomized clinical studies are needed to further assess the precise impact of anti-CD38 MoAbs on HSC and to determine the best mobilization strategy. A future review encompassing such studies could provide valuable insights into the topic and provide more definitive guidance on the optimal use of anti-CD38 MoAbs in patients undergoing mobilization and stem cell transplantation.

## 5. Conclusions

In summary, the existing literature indicates that anti-CD38 MoAbs interfere with HSC mobilization, collection, and engraftment. This interference likely results partly from direct toxicity on hematopoietic progenitors and partly from dysregulation of HSC adhesion to the bone marrow niche. Despite the higher incidence of poor mobilizers, the efficacy of PLX has been demonstrated in this context, enabling satisfactory yields of HSC, and granting access to ASCT for most patients. However, the optimal mobilization strategy for patients treated with anti-CD38 MoAbs remains undefined. Safety considerations should align with individual patient age and organ function, while potency must be tailored based on the planned number of ASCT(s) and the risk of poor mobilization. Given the unknown exact mechanism of interference, flexibility and salvage options are crucial for unexpected poor mobilization scenarios. Additionally, cost-effectiveness should be a priority. Further research is necessary to determine the best approach to stem cell mobilization in this patient population.

## Data Availability

The data presented in this study were derived from the following resources available in the public domain: PubMed (https://pubmed.ncbi.nlm.nih.gov/) accessed on 26 February 2024, Google Scholar (https://scholar.google.com/) accessed on 26 February 2024, Web of Science (https://www.webofscience.com/wos/woscc/basic-search) accessed on 28 February 2024, Scopus (https://www.scopus.com/search/form.uri?display=basic#researcher-discovery) accessed on 28 February 2024, ASH annual conference 2020 (Volume 136 Issue Supplement 1|Blood|American Society of Hematology (ashpublications.org)), ASH annual conference 2021 (Volume 138 Issue Supplement 1|Blood|American Society of Hematology (ashpublications.org)), ASH annual conference 2022 (Volume 140 Issue Supplement 1|Blood|American Society of Hematology (ashpublications.org)), ASH annual conference 2023 (Volume 142 Issue Supplement 1|Blood|American Society of Hematology (ashpublications.org)).

## Supplementary Materials

The following supporting information can be downloaded at: https://www.mdpi.com/article/10.3390/ph17070944/s1, Table S1: Mobilization outcomes in Zappaterra et al. [19].
