# Supplementary material for: Impact of Anti-CD38 Monoclonal Antibody Therapy on CD34+ Hematopoietic Stem Cell Mobilization, Collection, and Engraftment in Multiple Myeloma Patients—A Systematic Review
Source: Pharmaceuticals (Basel). 2024 Jul 15;17(7):944. doi: 10.3390/ph17070944 (PMC11280179; doi:10.3390/ph17070944)
Supplement: Supplementary file 1 [file pharmaceuticals-17-00944-s001.zip › pharmaceuticals-3032941-supplementary.pdf]

Table S1. Mobilisation outcomes in Zappaterra et al. [19]

	Daratumumab group N=20	Control group N=21	P value
Circulating CD34+ cells on 1st planned leukapheresis day, /mcL, median [IQR]	39,12 [24,59;52,57]	64,21 [53,88;101,12]	0,00048
CD34+ cells/mcL <20 on 1st planned leukapheresis day, n (%)	5 (25)	2 (10)	0,239
Plerixafor use, n (%)	4 (20)	1 (5)	0,18
1st day CD34+ cell yield, x106/kg, median (IQR)	3,98 [2,84;5,47]	6,87 [5,26;8,30]	0,00298

The U-Mann-Whitney and exact Fisher test were performed on the dataset provided by Zappaterra et al. [19]

Abbreviations: IQR: interquartile range; n: number.
